# Comparative Characterization of Bacterial Communities in Moss-Covered and Unvegetated Volcanic Deposits of Mount Merapi, Indonesia

**DOI:** 10.1264/jsme2.ME19041

**Published:** 2019-07-20

**Authors:** Annisa N. Lathifah, Yong Guo, Nobuo Sakagami, Wataru Suda, Masanobu Higuchi, Tomoyasu Nishizawa, Irfan D. Prijambada, Hiroyuki Ohta

**Affiliations:** 1 United Graduate School of Agricultural Science, Tokyo University of Agriculture and Technology 3–5–8 Saiwai-cho, Fuchu-shi, Tokyo 183–8509 Japan; 2 Ibaraki University College of Agriculture 3–21–1 Chuo, Ami-machi, Ibaraki 300–0393 Japan; 3 Department of Computational Biology, Graduate School of Frontier Science, The University of Tokyo Kashiwa Japan; 4 Department of Botany, National Museum of Nature and Science 4–1–1, Amakubo, Ibaraki Japan; 5 Graduate School of Biotechnology, University of Gadjah Mada Yogyakarta Indonesia

**Keywords:** volcanic deposit, early bacterial community, succession, *Betaproteobacteria*

## Abstract

Microbial colonization, followed by succession, on newly exposed volcanic substrates represents the beginning of the development of an early ecosystem. During early succession, colonization by mosses or plants significantly alters the pioneer microbial community composition through the photosynthetic carbon input. To provide further insights into this process, we investigated the three-year-old volcanic deposits of Mount Merapi, Indonesia. Samples were collected from unvegetated (BRD) and moss-covered (BRUD) sites. Forest site soil (FRS) near the volcanic deposit-covered area was also collected for reference. An analysis of BRD and BRUD revealed high culturable cell densities (1.7–8.5×10^5^ CFU g^−1^) despite their low total C (<0.01%). FRS possessed high CFU (3×10^6^ g^−1^); however, its relative value per unit of total C (2.6%) was lower than that of the deposit samples. Based on the tag pyrosequencing of 16S rRNA genes, the BRD bacterial community was characterized by a higher number of betaproteobacterial families (or genus), represented by chemolithotrophic *Methylophilaceae*, *Leptothrix*, and *Sulfuricellaceae*. In contrast, BRUD was predominated by different betaproteobacterial families, such as *Oxalobacteraceae*, *Comamonadaceae*, and *Rhodocyclaceae*. Some bacterial (*Oxalobacteraceae*) sequences were phylogenetically related to those of known moss-associated bacteria. Within the FRS community, *Proteobacteria* was the most abundant phylum, followed by *Acidobacteria*, whereas *Burkholderiaceae* was the most dominant bacterial family within FRS. These results suggest that an inter-family succession of *Betaproteobacteria* occurred in response to colonization by mosses, followed by plants.

The role of microbes in the formation of new soil, followed by the development of terrestrial ecosystems, is one of the main topics of general ecology and soil biology. In this respect, newly emplaced substrates by volcanic eruptions and the soil of retreated glacier areas have been examined as suitable target materials. Previous studies revealed that prior to plant colonization, microbes rapidly colonized newly exposed substrates and that their communities may have played roles in early biogeochemical processes and soil development (16, 25, 33, 35, 51). Kelly *et al.* (33) reported that 3–5 months after an eruption, Fimmvörðuháls lava flow (Eyjafjallajökull, Iceland) was colonized by a low-diversity microbial community dominated by *Betaproteobacteria*. This class of bacteria was related to non-phototrophic diazotrophs, such as *Herbaspirillum* spp., and chemolithotrophs, *e.g. Thiobacillus*. Several unvegetated, acidic volcanic deposits (3.5–6.0 years old) in the island of Miyake (Miyake-jima), Japan, harbored approximately 10^6^ cells (g dry weight^−1^) of autotrophic Fe (II) oxidizer communities represented by *Acidithiobacillus* spp. and *Leptospirillum* groups I, II, and III (16).

King (36) reported that reductant-rich volcanic deposits in hydrothermal and solfatara systems contained a number of chemolithotrophic bacteria, which were capable of exploiting high concentrations of inorganic substrates, particularly sulfides. This is the case for Fimmvörðuháls lava flow, in which early bacterial colonizers were found to be related to galena (PbS)- and H_2_S-oxidizing *Thiobacillus plumbophilus* (33). Chemolithotrophy primarily occurs in reductant-poor volcanic ecosystems, in which facultative chemolithotrophs are known to oxidize CO, H_2_, or both for energy (35, 36). The study of a 1959 cinder deposit in Hawaii revealed a diverse community that comprised *Cyanobacteria*, *Acidobacteria*, and *Alphaproteobacteria* along with microorganisms that were specifically capable of CO and hydrogen oxidation when pioneering the deposit (65). The contribution of atmospheric trace gases in primary production was also reported for Antarctic desert soil (28). Furthermore, H_2_-oxidizing, facultative chemolithotrophic bacteria, *e.g. Cupriavidus pinatubonensis* and *C. laharis*, represented the main culturable bacterial population in the volcanic mudflow deposit of Mt. Pinatubo (Philippines) (51, 52).

Microbial succession across post-eruption chronosequences occurs well before plant colonization. A metagenomic analysis from an unvegetated site of Miyake-jima volcanic deposits (3.5–9.5 years old) revealed the abundance of a nitrogen fixation gene (*nifH*). This contributed as the main feature of the deposits and accounted for approximately 50% of nitrogen-cycling related genes. Hence, the composition of N_2_-fixing bacteria shifted from an obligate chemolithotrophic Fe (II)-oxidizing community (53) to heterotrophic and/or facultative lithotrophic diazotrophs, such as *Burkholderia*, *Beijerinckia*, and *Leptothrix* (17). In addition to new volcanic substrates, early microbial succession is well characterized in unvegetated and recently deglaciated soils (49, 54, 70). A study on pre-plant stages (primary succession) in deglaciated soils of the high-Andean chronosequence showed that barren soils were colonized by a diverse community of *Cyanobacteria*. Moreover, a significant increase in cyanobacterial diversity corresponded with marked increases in the heterotrophic microbial biomass and soil enzyme activity (54). Furthermore, soil nitrogen fixation rates increased by almost two orders of magnitude during the first 4–5 years of succession and many years before the establishment of mosses, lichens, or vascular plants. The relative abundance of cyanobacterial populations increases with soil age, and its correspondence with the accretion of soil nitrogen pools in unvegetated soils also holds true for the emerging landscape near the receding Puca Glacier (southeast Peru) (49).

Microbial succession on newly exposed, unvegetated substrates is an important factor for early biogeochemical cycling and ecosystem development. However, plant colonization significantly alters the pioneer microbial community composition through carbon inputs in the form of root exudates and litter deposits (2, 20), which further facilitates ecosystem development. Glacial retreat areas have hosted many studies to examine shifts in microbial community structures in association with colonizer plants during early primary succession (6, 37). In the context of these studies, the trajectories of bacterial succession were generally selective shifts in *Acidobacteria* and *Alphaproteobacteria*. The population levels of these groups were low in the case of young glacial soils, but relatively abundant in vegetated soils. However, an opposite shift was recorded in the case of *Betaproteobacteria*. A study on actively retreating glaciers (6) reported significant differences in bacterial community compositions between the two glacial sites regardless of the successional stage. Despite differences in the geography, climate, and soil characteristics (chemical and physical) of the two sites, the soil bacterial community structure was observed to converge during plant succession. Across the successional stages, differences in community compositions were related to shifts in the relative abundances of specific bacterial phyla and sub-phyla. Compositional shifts at the Easton glacier reflected a decrease in initially abundant *Betaproteobacteria* with an increase in *Acidobacteria*, *Bacteroidetes*, and *Verrucomicrobia*. However, initially abundant candidate division WPS at the Mendenhall glacier declined during succession, whereas *Acidobacteria* increased in relative abundance.

To the best of our knowledge, only a few studies have investigated the relationship between pioneer plant colonization and microbial communities with respect to volcanic environments. One study examined scoria deposits in a sub-alpine desert on Mt. Fuji (Japan) using phospholipid fatty acid, denaturing gradient gel electrophoresis, and Biolog microplate analyses (69). The findings obtained showed a marked shift in the microbial community structure as a consequence of initial colonization by the pioneer herb, *Polygonum cuspidatum* followed by *Larix kaempferi* into the central areas of island-like communities. Another study that focused on the recent Miyake-jima volcanic deposits along a vegetation gradient (23) revealed using 16S rRNA pyrosequencing that the abundance of the *Oxalobacteraceae* family of *Betaproteobacteria* and *Xanthomonadaceae* family of *Gammaproteobacteria* increased in response to changes in the vegetation cover from grass to shrubs.

Mt. Merapi, an active stratovolcano of Indonesia, last erupted in 2010, which affected all areas around the mountain (5). The affected area, three years after the eruption, showed the appearance of moss-covered patches in volcanic deposits and, thus, provided a unique study site for clarifying the relationship between pioneer moss colonization and microbial communities. In the present study, samples were collected at moss-covered and unvegetated sites and analyzed for their bacterial community structures by the tag pyrosequencing of 16S rRNA genes. The results obtained provide insights into the succession of betaproteobacterial groups in response to moss colonization and facilitate our understanding of the relationship between early soil genesis and bacterial community.

## Materials and Methods

### Site description and sampling

Mt. Merapi, located near central Java (Indonesia), is an area of 300–400 km^2^ at 2,978 m above sea level (m.a.s.l.) (Fig. S1). Eruptions during the 20^th^ century that typically recurred every 4–6 years produced viscous lava domes that collapsed to form pyroclastic flows and subsequent lahars. These eruptions were relatively small, with eruptive volumes of 1–4×10^6^ m^3^ and magnitude/volcanic explosivity indices of 1–3 (7, 57). The eruption in 2010 affected all areas around Mt. Merapi. Pyroclastic flow reached 4 km in the north, 11.5 km in the west, 7 km east, and approximately 15 km in the south reaching the Kaliadem area. Explosive bombs from the summit went in all directions (approximately 4 km) and emitted large amounts of ash and gas into the atmosphere (10, 68). Volcanic ash deposits derived from the eruption were characterized by high contents of SiO_2_ (52.6–60.3 wt %), K_2_O (1.99–2.99 wt %), and Al_2_O_3_ (16.6–18.9 wt %) (68).

Our study sites were established on volcanic deposit-covered land (sites BR and BRU) and a forest site (FR). FR, along the Bebeng river at the foothills of Mt. Merapi, was selected as a reference site (Fig. S1). Site BR (07°34′39″ S, 110°26′53″ E, and 1,181 m.a.s.l.) was not vegetated. Site BRU (07°34′39″ S, 110°26′51″ E, and 1,199 m.a.s.l.) was majorly inhabited by *Campylopus umbellatus* (moss) and partly by *Athyrium* sp. (lady fern), *Imperata cylindrica* (cogon grass), and *Anaphalis javanica* (Javanese edelweiss). Reference soils were sampled from site FR (07°34′43″ S, 110°26′51″ E, and 1,199 m.a.s.l.), which was predominantly vegetated by *Acacia decurrens* (acacia), *Schima wallichii* (puspa), and *Pennisetum purpureum* (elephant grass). Samples were collected on March 6, 2013 from three points (depth of 30 cm) at a 1-m interval (Fig. S1). Samples were divided into two portions (in sterile plastic bags) and kept on ice. One sample bag was stored at 4°C and the other at –20°C for later bacteriological analyses (within one week of sampling) and DNA extraction, respectively. Roots and debris from forest soil samples (FRS) were removed prior to these analyses and DNA extraction.

### Chemical analysis and plate counts

The total carbon (TC) and total nitrogen (TN) contents of the samples were measured using an NC analyzer (SUMIGRAPH NC-22F; Sumika Chemical Analysis Service, Tokyo, Japan). pH was assessed using the mass ratio 1:2.5 (sample: water). The volumetric water content was analyzed by drying the material at 105°C overnight. Regarding the bacterial plate count, nitrogen-free Ashby’s mannitol medium (pH 7.2) was used, containing (per L): mannitol (15 g), CaCl_2_·7H_2_O (0.2 g), K_2_HPO_4_ (0.2 g), MgSO_4_·7H_2_O (0.2 g), 10% (w/v) MoO_3_ (0.1 mL), 10% (w/v) FeCl_3_ (0.05 mL), and agar (15 g). Sample dilutions plated in duplicate were incubated at 30°C.

### DNA extraction

Total DNA was extracted in duplicate from 0.5–1.0 g of samples using ISOIL for Bead Beating (Nippon Gene, Tokyo, Japan) with skim milk powder (Wako, Osaka, Japan) according to the manufacturer’s instructions with slight modifications (50). Additionally, for volcanic deposit samples from sites BR and BRU, DNA was extracted by the CTAB (hexadecyltrimethylammonium bromide) method (71) as described in our previous studies (16, 17, 23). Duplicates of extracted DNA were pooled together and subjected to molecular analyses.

### Quantification of 16S rRNA gene abundance

The gene abundance of bacterial 16S rRNA was assessed by fluorescent quenching-based, real-time qPCR (40) using the primers Q-10f (5′-AGTTTGATCCTGGCTCAG-3′) and 907R (5′-CCGT CAATTCCTTTRAGTTT-3′) as described previously (50). The 5′-end fluorescence-labeled primer, Q-10f, was purchased from J-Bio21 (Kisarazu, Japan). The PCR mixture (30 μL) was composed of 2 μL of template soil DNA, 0.5 μL of 10 pmol μL^−1^ primers, Ex Taq polymerase, dNTPs, and 3 μL of optimized 10× Ex buffer (Takara Bio, Kusatsu, Japan). qPCR was conducted on a PCR iCycler apparatus (Bio-Rad, Hercules, CA, USA) with the thermal cycle program of an initial cycle of 95°C for 2 min, 45 cycles of 95°C for 30 s, 52°C for 45 s, and 72°C for 1.5 min. The intensity of fluorescence quenching was measured to calculate the quenching rate as described by Kurata *et al.* (40). A standard curve was generated using the total genomic DNA of *Escherichia coli* and the copy number of 16S rRNA genes was calculated using the URI Genomics & Sequencing Center program (https://cels.uri.edu/gsc/cndna.html).

### 16S rRNA-based tag-pyrosequencing

The V1–V2 region of the 16S rRNA gene (16S rDNA) was amplified using the universal primers 27Fmod and 338R. Amplification conditions were set at 96°C for 2 min, 20 cycles of 96°C for 30 s, 55°C for 45 s, and 72°C for 1 min with a final extension of 72°C for 10 min on a 9700 PCR system (Life Technologies Japan, Tokyo, Japan) according to the protocol of Kim *et al.* (34). PCR products were confirmed by 2% agarose gel electrophoresis, purified by the Agencourt AMPure XP PCR purification kit (Beckman Coulter, Brea, CA, USA), and quantified using a Quant-iT PicoGreen dsDNA Assay Kit (Thermo Fisher Scientific, Waltham, MA, USA). A composite sample was prepared by pooling approximately equal amounts of the PCR amplicons from each sample. This was followed by pyrosequencing using Roche 454 GS FLX Titanium or 454 GS Junior (Roche Applied Science, Penzberg, Germany), according to the manufacturer’s instructions.

### Sequence data processing and analysis

The sequence data obtained from 454-pyrosequencing reads were analyzed using the pipeline developed by Kim *et al.* (34). In short, the raw sequence data obtained from 454 pyrosequencing was initially assigned to each sample on the basis of their barcode sequence. Reads with an average quality value <25 and those without both of the universal primer sequences were then filtered off. A total of 28,585 reads were ultimately recovered from raw data. In further analyses, 2,000 reads were randomly selected from each library. To define operational taxonomic units (OTUs), the clustering of 16S rDNA reads was performed with the UCLUST program (https://www.drive5.com/) using 96% pairwise identity as the cut-off. Representative sequences of each OTU were assigned a bacterial genus using a BLAST search (with 96% pairwise identity as the cut-off) against the Ribosomal Database Project (RDP, v10.27) and the reference genome database. The latter was constructed by adding 1,482 complete and 506 draft bacterial genomes from the NCBI FTP site (ftp://ftp.ncbi.nlm.nih.gov/) (34). OTUs, classified as the taxonomic information of a representative sequence with a high pairwise identity, were obtained by comparisons between the two databases. The weighted UniFrac distance was calculated from the phylogenetic tree of the representative sequences of an OTU by the PyCogent software library (38).

### Statistical analysis

The properties (chemical and biological) and influence of the sampling site on bacterial diversity indices (Shannon, Inverse Simpson, ChaoI, and OTU number) were evaluated using ANOVA with Tukey’s honestly significant difference (HSD) test to assess the significance of differences and clarify the rank order. Significance was defined at *P*<0.05. Similarities and differences in the bacterial community structure among the samples were examined using the weighted UniFrac distance with a principal coordinate analysis (PCoA) ordination technique. To assess whether a significant difference exists between CTAB-extracted and ISOIL-extracted communities, an analysis of molecular variance (AMOVA) in Mothur was performed using the sub-dataset of BRD and BRUD samples by two DNA extraction methods. A canonical correspondence analysis (CCA) was performed to examine the relationship between bacterial communities and environmental variables. The percentage abundance of bacterial families in each volcanic deposit and the soil sample library was used as the species input. The vegetation type (unvegetated and *C. umbellatus* moss) and chemical properties (pH, TC, TN, C:N ratio, and water content) served as environmental inputs. Ordination plots of CCA results were performed using the function cca from the R package ‘vegan’ (http://cran.r-project.org/web/packages/vegan/index.html). The relationship between each environment variable and bacterial communities was investigated using the function anova.cca from the R package ‘vegan’ to assess the significance of constraints.

### Phylogenetic tree

The 16S rRNA gene sequences of OTUs were compared with those of the OTUs and type strains in the RDP and NCBI databases. Clustal W was used to perform a multiple sequence alignment. Phylogenetic trees were reconstructed in MEGA version 7 using neighbor joining with 1,000 bootstrap replicates. Genetic distances were calculated using the maximum composite likelihood model.

### Sequence data accession number

Filtered pyrosequencing reads were deposited in the DDBJ Sequence Read Archive database under the accession number DRA002857.

## Results

### Chemical characteristics, plate count of bacteria, and abundance of the 16S rRNA gene

The chemical properties of volcanic deposit samples from sites BR (BRD), BRU (BRUD), and FR (FRS) are shown in Supplementary Table S1. The pH values of BRD and BRUD were similar to that of FRS and varied between 7.2–7.5. The TC and TN contents of BRD and BRUD were lower than those of FRS in three to four and two orders of magnitude, respectively (Table S1). Ashby’s mannitol medium was used to estimate the population level of bacteria that was able to grow in nitrogen-free medium. Plate counts were approximately 1.7×10^5^ CFU g^−1^ for BRD and 8.5×10^5^ CFU g^−1^ for BRUD, which were markedly lower than that of FRS (3.0×10^6^ CFU g^−1^). The quantification of 16S rRNA gene abundance supported the lower bacterial population density in BRD and BRUD than in FRS: 7.2 and 3.6×10^8^ gene copies g^−1^ for the BRD and BRUD samples, respectively, vs. 8.0×10^10^ gene copies g^−1^ for the FRS sample. The gene abundance of 16S rRNA in BRD and BRUD was similar to that ([2.64±1.70]×10^8^ gene copies g^−1^) at the bare sites of Hawaiian volcanic deposits (66).

### Similarity and diversity of bacterial communities

**Fig. 1:**
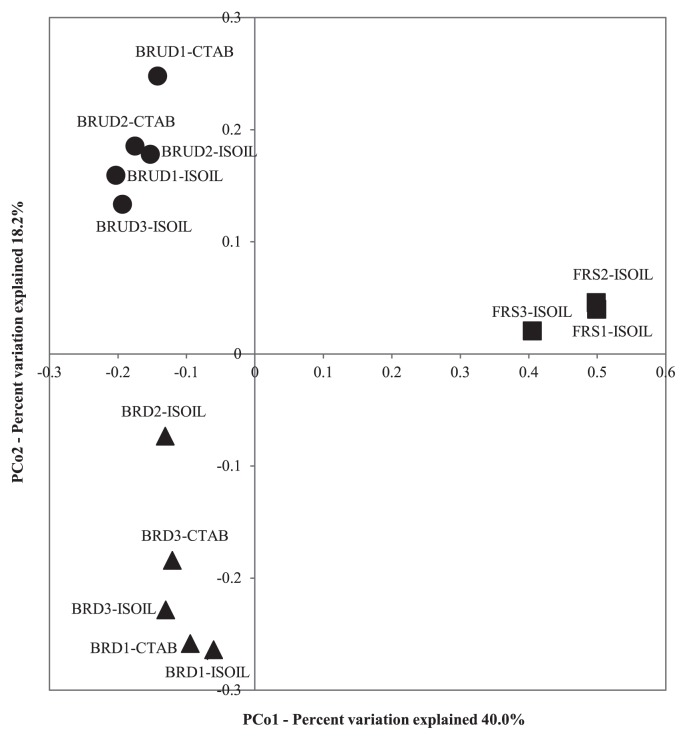
Principal coordinate analysis (PCoA) plots of bacterial communities of volcanic deposit (▲, ●) and soil (■) samples by weighted UniFrac.

Nearly 100% (99.98%) of 26,000 reads from 13 sample libraries (constituted by 9 ISOIL-DNA samples of BRD, BRUD, and FRS triplicates and 4 CTAB-DNA samples: two out of BRD and BRUD triplicates) were annotated and assigned bacterial phyla. PCoA was performed on the 13 libraries to estimate similarities or differences among the bacterial communities. The results obtained and shown in Fig. 1 indicate the distinct clustering of bacterial communities across samples. When tested by AMOVA, no significant differences were observed between the ISOIL-DNA and CTAB-DNA libraries of BRD and BRUD (*P*=0.448). Therefore, ISOIL-DNA libraries were selected for subsequent analyses. A total of 18,000 reads were classified into 2,879 OTUs. The average number of OTUs recorded for the bacterial communities of BRD, BRUD, and FRS were 397, 287, and 751, respectively; suggesting the bacterial community diversity in BRUD to be the lowest (Table S2). These results were supported by the Shannon, Inverse Simpson, and ChaoI indices calculated from pyrosequencing data (Table S2) and rarefaction curves (Fig. S2).

### Comparison of the bacterial community structure

**Fig. 2:**
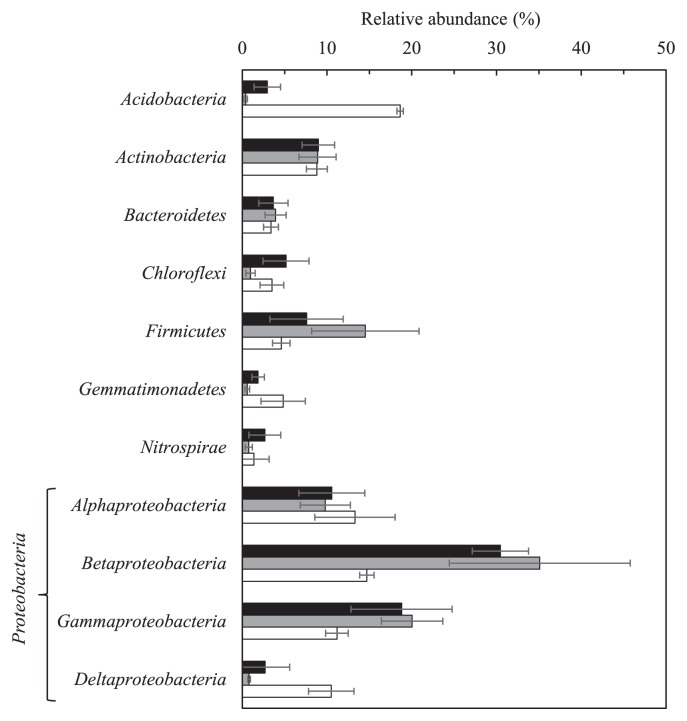
Relative abundance of major phyla and classes of *Proteobacteria* in volcanic deposits (■, BRD; 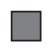, BRUD) and the forest soil (□). Error bars indicate standard deviations (*n*=3).

*Proteobacteria* was the most abundant phylum observed in the bacterial community of FRS (mean percent relative abundance of triplicate samples: 50%), BRD (63%), and BRUD (66%) (Fig. 2). *Firmicutes* was the second most abundant group in the BRD and BRUD communities (8 and 15%, respectively), which was followed in order by *Actinobacteria* (9% for both). On the other hand, *Acidobacteria* (19%) and *Actinobacteria* (9%) constituted the second major groups in the FRS community. *Betaproteobacteria* was the most prominent bacterial class of *Proteobacteria* found in the BRD (31%) and BRUD (35%) communities. The relative abundances of alpha-, beta-, gamma-, and deltaproteobacterial classes (11–18%) were observed in the FRS community (Fig. 2).

Comparisons of minor bacterial phyla (relative abundance: <2%) also indicated differences between the BRD/BRUD and FRS bacterial communities. The *Deinococcus-Thermus* group was only found in the BRD and BRUD communities. The phylum *Cyanobacteria* was present at a relatively higher abundance in the BRD and BRUD (0.8–2%) communities than in the FRS community (0.1–0.4%). The abundance of the phylum *Thermotogae* appeared to be higher in the BRUD community (1.5%) than in the BRD and FRS communities (0.2%).

**Fig. 3:**
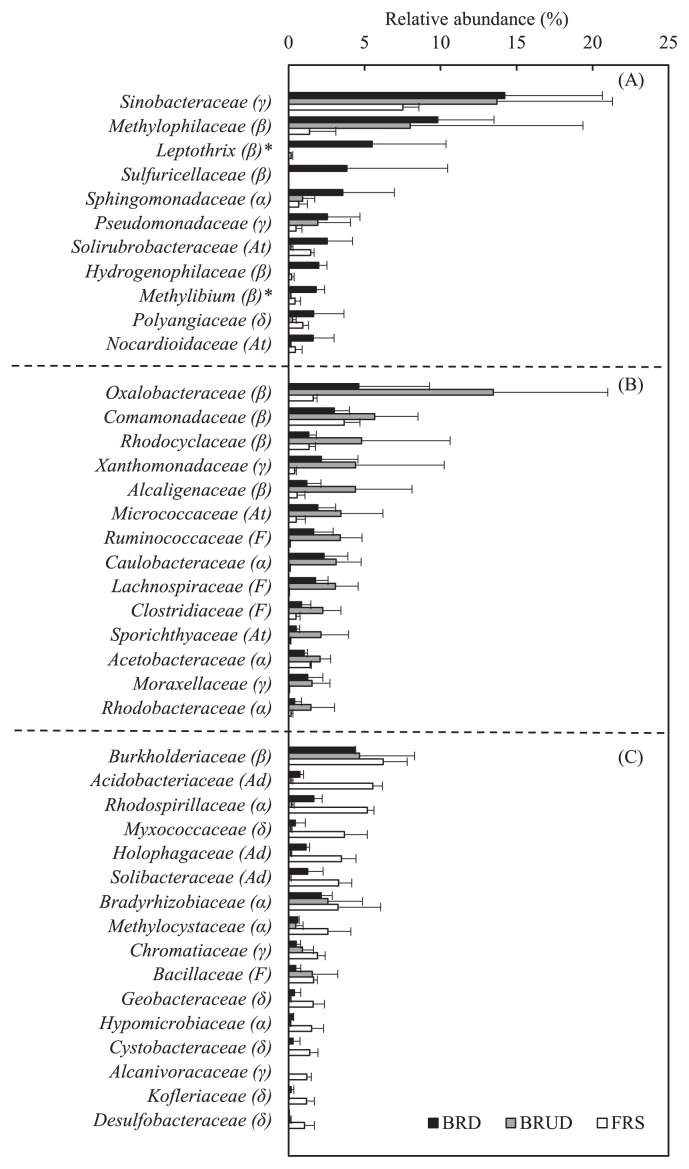
Major families with relative abundance greater than 1%. Grouping of the top 11, 14, and 16 families in the bacterial communities of BRD (A), BRUD (B), and FRS (C), respectively. The family name is followed by letter(s) in parentheses, showing the phyla *Acidobacteria* (*Ad*), *Actinobacteria* ( *At*), and *Firmicutes* ( *F*), and the classes *Alphaproteobacteria* (*α*), *Betaproteobacteria* (*β*), *Gammaproteobacteria* (*γ*), and *Deltaproteobacteria* (*δ*). *Burkholderiales inserta sedis.

Further examinations of bacterial OTUs revealed that 89.5% were assigned to 189 known families. Fig. 3 shows the major bacterial families (41 families; with relative abundance >1%), comprising the top 11, 14, and 16 families of the BRD, BRUD, and FRS communities, respectively. The BRD bacterial community was characterized by a high relative abundance of *Methylophilaceae*, *Leptothrix* (*Burkholderiales incertae sedis*), and *Sulfuricellaceae* of the class *Betaproteobacteria*; accounting for 9.8, 5.5, and 3.8%, of total OTUs, respectively. The family *Sinobacteriaceae* of the class *Gammaproteobacteria* represented the most prominent group not only in BRD (14.2%), but also in BRUD (13.7%) and FRS (7.5%) (Fig. 3A). The predominant *Betaproteobacteria* of the BRUD bacterial community were the members of *Oxalobacteraceae* (13.5%), *Comamonadaceae* (5.7%), and *Rhodocyclaceae* (4.8%), which were different from those in the BRD community (Fig. 3B). In contrast to the BRD and BRUD communities, the FRS bacterial community was characterized by the appearance of *Acidobacteria* families, such as *Acidobacteriaceae* (5.6%), *Holophagaceae* (3.5%), and *Solibacteraceae* (3.3%) (Fig. 3C). *Rhodospirillaceae* (5.2%) belonging to the class *Alphaproteobacteria* and *Myxococcaceae* (3.7%) of the class *Deltaproteobacteria* were specifically abundant in the FRS community. In contrast, the family *Burkholderiaceae* of *Betaproteobacteria* was present in BRD and BRUD (4.4–4.7%) and also in FRS communities (6.2%). Similar results were observed for the family *Bradyrhizobiaceae* (*Alphaproteobacteria*) with 2.2, 2.6, and 3.3% abundance in the BRD, BRUD, and FRS bacterial communities, respectively.

### Relationship between the bacterial community and environment factors

**Fig. 4:**
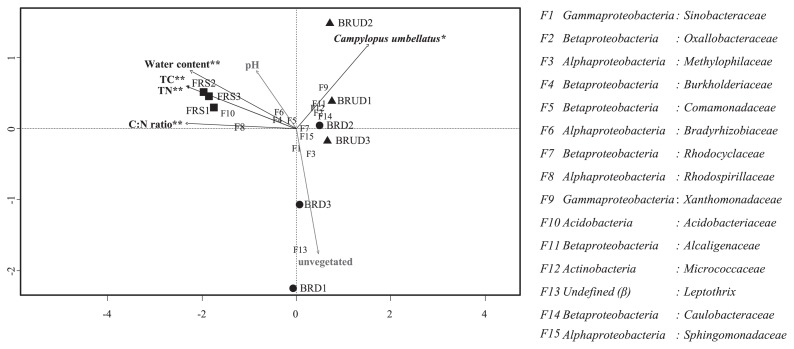
Canonical correspondence analysis (CCA) ordination plots of bacterial communities of volcanic deposits (▲, ●) and results of the analysis of environmental factors affecting bacterial distribution. The direction of the arrows indicates an increasing value of environmental parameters and the arrow length indicates the degree of the correlation with the represented axis. The numbers correspond to bacterial families (given in the key on the right), which are ranked according to their abundance. The asterisk symbol (*, 0.01<*P* value<0.05; **, 0.001<*P* value<0.01) represents a correlation between the environmental factor and bacterial community, tested by ANOVA.

CCA was performed to reveal possible links between environmental factors and known bacterial families. The statistical test by ANOVA showed correlations between water content, TC, TN, and the C:N ratio and bacterial communities. The relationship between environmental factors and specific bacterial families was shown in Fig. 4. Positive correlations between *Oxalobacteriaceae*, *Xanthomonadaceae*, *Micrococcaceae*, *Alcaligenaceae*, and *Caulbacterceae* and the moss, *C. umbellatus*, were observed in site BRUD, whereas *Rhodospirillaceae* and *Acidobacteriaceae* correlated with increases in TC, TN, the C:N ratio, and water content in site FRS. However, pH did not significantly affect bacterial families in the present study sites.

## Discussion

Newly exposed volcanic deposits served as habitats for microorganisms in spite of lacking even detectable amounts of organic matter and vegetation (16, 19, 25, 35, 47). To the best of our knowledge, bare volcanic deposits (3 months-8.5 years old) harbor 10^6^–10^8^ g ^−1^ of bacteria (direct cell count) and approximately 10^6^ g ^−1^ of culturable bacteria (16, 33). Our plate count data (10^5^ g^−1^ for BRD) was consistent with previous findings and suggested that bacterial colonization was not strictly dependent on carbon and nitrogen availabilities in deposits. In other words, a deficiency in organic nutrients may result in the significant occurrence of chemolithotrophy, thereby yielding a fully detectable bacterial biomass, as described by King (36). Alternatively, microbial phototrophy may occur in new terrestrial substrates, as demonstrated in recently deglaciated soils that harbored *Cyanobacteria* as pioneer microbes (32, 45, 54). However, in our BRD and BRUD samples, the population level of *Cyanobacteria* was as low as 0.3–0.4%. Very low levels of *Cyanobacteria* have also been found in previously examined volcanic deposits (16, 23, 25, 33, 35). The reasons for this low cyanobacterial abundance currently remain unclear.

The bacterial community of the unvegetated Mt. Merapi volcanic deposit (BRD) was predominated by *Betaproteobacteria* (Fig. 2). The major families of this class with relative abundance of more than 3% were as follows: *Methylophilaceae* (9.8%), *Leptothrix* (family *Burkholderiales incertae sedis*) (5.5%), and *Sulfuricellaceae* (3.8%) (Fig. 3A). Further examinations revealed that the majority of OTUs of *Methylophilaceae* belonged to the genus, *Methylotenera* (Fig. S3). The genus *Methylotenera* comprises two species: *Methylotenera mobilis* (29) and *M. versatilis* (30). *M. mobilis* is described as an obligate methylamine-utilizing bacterium, whereas *M. versatilis* is a facultative methylotroph. The type strains of these species were isolated from the sediments of Lake Washington, USA. using media supplemented with methylamine. In nature, methylamine is found in the breakdown of dead animals or plant matter; however, it is not expected to exist in organic matter-poor volcanic deposits. Interestingly, Eyice *et al.* (14), by combining stable isotope probing and metagenomics, identified *Methylotenera* and *Thiobacillus* as the main consumers of dimethylsulfide (DMS), an atmospheric trace gas present in terrestrial environments. Annual DMS emissions into the atmosphere from terrestrial ecosystems are estimated to be 3.8 Tg (64). As described by King (36), volcanic deposits lacking significant levels of organic matter represent a habitat that may harbor chemolithotrophic trace gas oxidizers, which are capable of deriving substrates for energy generation from the atmosphere. Therefore, the occurrence of *Methylophilaceae* and *Hydrogenophilaceae*, including *Thiobacillus*, in the BRD community will be explained by their growth at the expense of atmospheric DMS as a carbon and energy source.

The OTU (OTU00084) of *Sulfuricellaceae* (relative abundance: 3.8% in BRD) is related to the cluster of the genus *Sulfuriferula*, a sulfur-oxidizing, chemolithotrophic bacterium (62). The sequence of this OTU (304 nucleotides length) is identical to the partial sequence of the uncultured betaproteobacterium clone, OY07-C004, which was recovered from a seven-year-old Miyake-jima volcanic deposit (16) (Fig. S3). This finding suggests that this OTU group is a common and early colonizer of the volcanic environment. The genus *Sulfuriferula* has so far been represented by only three species: *S. multivorans*, *S. plumbophilus* (formerly *T. plumbophilus*), and *S. thiophila*. Amongst them, *S. thiophila* may grow using hydrogen as the electron donor (63) and, thus, is expected to exist by consuming atmospheric H_2_ (mixing ratio: 550 ppbv) (9) in carbon-poor environments, as observed in Mt. Kīlauea volcanic deposits (35). As shown in Fig. S3, OTU00134 (relative abundance: 4.3%) specific to BRD was closely related to the sequence of the arsenite-oxidizing bacterium, *Leptothrix* sp. S1.1, which was isolated from the settling pond sediments of As- and Fe-containing mine-drainage water (3). However, we do not have any data on As (III) or Fe (III) oxidation in Mt. Merapi volcanic deposits.

The family *Sinobacteriaceae* (*Gammaproteobacteria*) was the most abundant group in the BRD community (Fig. 3A). In the phylogenetic tree of *Sinobacteriaceae* (Fig. S4), sequences of the major OTUs (OTU00073 and OTU01094) clustered with the genus, *Nevskia*. One of them (OTU00073) showed similarity to the partial sequence of *Nevskia* sp. KP1-11, isolated from an unvegetated volcanic deposit (at a distance of 20 m from the seashore) in the south of Miyake-jima (46). *Nevskia ramosa* isolates grew at the air-water interface and benefitted by trapping ammonia from the air despite the absence of nitrogenase activity (18). This may explain the presence of *Nevskia* in unvegetated volcanic deposit(s). The most abundant sequence (OTU00047; relative abundance: 9.7% in BRUD community) in *Sinobacteriaceae* clustered with an uncultured bacterium clone (SC6-RK103) that was recovered from white areas in the mural paintings of Etruscan tombs (11). This clone was annotated as *Panacagrimonas perspica* (26), isolated from soil sampled from a ginseng field with an identity of 97%. No further information is available for OTU00073.

An analysis of the BRUD bacterial community revealed betaproteobacterial succession from *Leptothrix* and *Sulfuricellaceae* to *Oxalobacteraceae*, *Comamonadaceae*, and *Rhodocyclaceae*. However, *Methylophilaceae* was still predominant (8%) in BRUD (Fig. 3B). The most abundant bacterial family in BRUD, *Oxalobacteraceae*, accounted for 13.7% of the total community, which was reported to harbor root-colonizing, heterotrophic bacteria in a succession of bacterial communities during early plant development (21, 22). Similarly, a positive correlation (*P* value, 0.04) between *Oxalobacteraceae* and *C. umbellatus* (moss) was noted in the present study, as shown by CCA with the ANOVA test (Fig. 4). The phylogenetic analysis of *Oxalobacteraceae* OTUs from BRUD in the present study revealed that the major OTU (OTU00008) accounted for 9.6% of the BRUD community and was clustered within the genus *Massilia* that included the following: *M. timonae* R2-11, *M. plicata* LB-U, and *M. dura* 16^T^ (Fig. S4). Amongst these, *M. timonae* R2-11 and *M. plicata* LB-U were described as moss-associated bacteria (59). The second major OTU (OTU00143; relative abundance: 1.6%) was closely related to *Undibacterium oligocarboniphilum* EM 1^T^; a novel bacterium, capable of growing in low carbon substrate concentrations (13) (Fig. S4). Another OTU (OTU00091, 0.7%) was clustered with weathering-associated *Janthinobacterium agaricidamnosum* NBRC 102515^T^ (43, 60, 61) (Fig. S5).

In ecological succession, mosses are the pioneering species and important drivers of the biogeochemical cycles of the ecosystem (1, 41, 55). Mosses contribute carbon to support soil and also provide a microhabitat for a wide diversity of microorganisms (44). *Campylopus*, a moss, has been reported as the pioneer vegetation of volcanic areas (4, 8, 42). Jackson (27) reported that mosses, presumably with the assistance of associated microorganisms, strongly enhanced the chemical weathering of granitic genesis on which they were growing and also mediated the formation of soil containing various biogenic secondary minerals. This may have occurred in the BRU site due to the association of *Campylopus* (moss) with the above-described bacteria (*Massilia*). Weathering through the moss-bacterium relationship warrants further study.

The occurrence of OTUs affiliated with *Alcaligenaceae* and *Rhodocyclaceae* (OTU00631 and OTU00228, respectively) cannot be explained as simply as that of *Oxalobacteraceae* OTUs. OTU00631 is closely related to the uncultured bacterium clone C SML 53 (Fig. S5), which was recovered from the surface microlayer of the water column in Coldspring Lake, Canada (12). The latter is a small (<1 km^2^), shallow (average depth ~3 m, maximum depth ~7 m), wetland-associated, highly organic, and Fe-rich soft water Canadian Shield lake. The surface microlayer of the lake was assumed to harbor arsenite-, Fe (II)-, and thiosulfate-oxidizing bacteria. Similarly, the sequence of OTU00228 in *Rhodocyclaceae* was related to the partial sequence of the uncultured bacterium clone SX2-1 from a sample of Fe- and S-containing wasted bioleaching liquid (pH 3.0) (Fig. S5) (24). In relation to these sulfur-rich environments, it is noteworthy that OTU00228 is grouped with the clade of *Sulfuritalea hydrogenivorans* DSM 22779^T^, which grows chemolithoautotrophically under anoxic conditions by oxidizing reduced sulfur compounds and hydrogen (39) (Fig. S5). These findings suggest that bacteria corresponding to *Alcaligenaceae* and *Rhodocyclaceae* OTUs perform chemolithotrophic metabolism.

The FRS bacterial community is predominated by the families of *Acidobacteria*, *Alphaproteobacteria*, *Gammaproteobacteria*, and *Deltaproteobacteria*. No other *Betaproteobacteria*, except for the most abundant family *Burkholderiaceae* (6.2%), was ranked in the top 14 families. *Burkholderiaceae* was also one of the major members in the BRD (4.4%) and BRUD (4.7%) communities. To compare the lower taxonomic level of *Burkholderiaceae* OTUs among the three sites, a phylogenetic tree analysis was conducted for OTUs with a relative abundance of >0.1% at any of the sites (Fig. S6). Amongst the 20 OTUs examined, 9 were only found in FRS, whereas 4 were specific to BRD and/or BRUD. Two OTUs were detected in all sites and the others were found in BRD+FRS or BRUD+FRS. This result suggests that approximately half of the members of *Burkholderiaceae* were new when appearing during the change from volcanic deposits to soil. The sequence of OTU00241, specific to BRD (relative abundance: 0.2%) and BRUD (2.6%), is identical to the partial 16S rRNA gene sequence of *Ralstonia* sp. ML7 that was isolated from soil and was able to degrade the aliphatic polyesters, poly (ɛ-caprolactone) and poly (hexamethylene carbonate) (58). The probable existence of such a polyester-degrading bacterium in volcanic deposits cannot currently be explained. However, this may be expected based on previous findings showing the isolation of 4-dichlorophenoxyacetic acid (2,4-D)-degrading bacteria from pristine environments with no history of 2,4-D exposure (31). Weber and King (67) isolated novel CO-oxidizing *Paraburkholderia* and *Burkholderia* from Mt. Kīlauea volcanic deposits (Hawaii). Our Merapi OTUs were not clustered with them (Fig. S6).

**Fig. 5:**
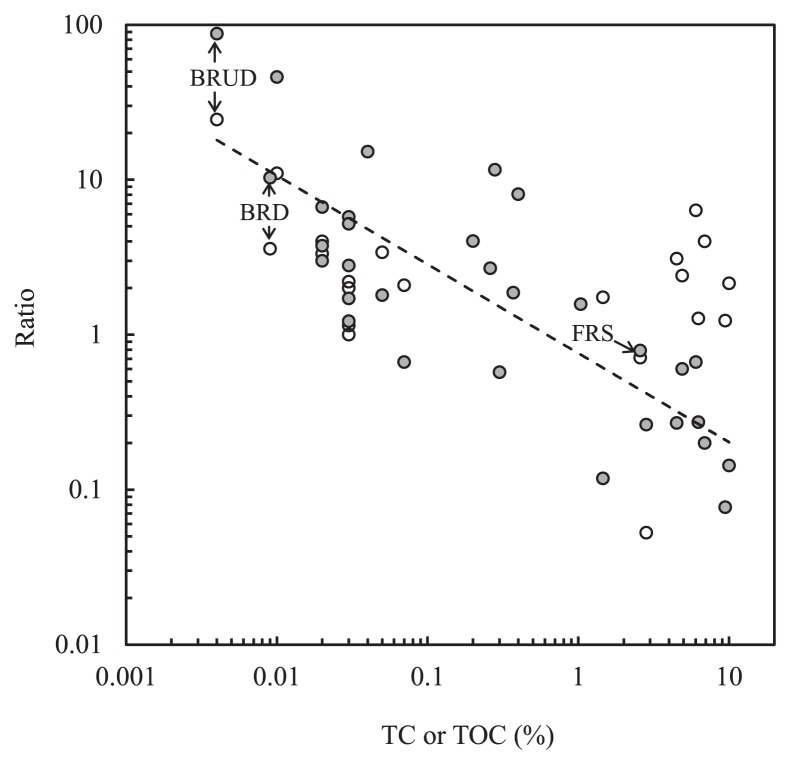
Ratios of *Alphaproteobacteria* to *Acidobacteria* (○) and *Betaproteobacteria* to *Acidobacteria* ( 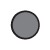) in bacterial communities of volcanic deposits and environmental soils. The plots of this study (BRD, BRUD, and FRS) are shown with arrows. The other plots were calculated from the data of Castle *et al*. (6), Fujimura *et al*. (16), Guo *et al*. (23), and Summers *et al*. (56). The relationship between TC or TOC (total organic carbon) and the ratio of *Betaproteobacteria* to *Acidobacteria* is approximated as a broken line: *y*=0.76 *x*^−0.58^ (*r*^2^=0.661).

The phylum *Acidobacteria* is one of the most abundant soil bacteria and *Acidobacteria* subgroups 1, 3, 5, and 6 collectively account for 87% of the total *Acidobacteria* community in forest soils of the western Brazilian Amazon (48). The majority of acidobacterial OTUs in FRS belonged to *Acidobacteria* subgroup 1 (Gp 1) (Fig. S7). Fierer *et al.* (15) proposed oligotrophic and copiotrophic phyla based on the findings of molecular analyses for a total of 71 unique soils, representing soil and site characteristics in the USA. In their study, bacteria belonging to *Acidobacteria* were the most abundant in soils with very low resource availability (oligotrophic attributes). However, the relative abundances of *Betaproteobacteria* and *Bacteroidetes* were the highest in soils with high carbon availability (copiotrophic attributes). In their discussion citing autotrophic ammonia oxidizers in *Betaproteobacteria*, every member of *Acidobacteria* and *Betaproteobacteria* was not distinctly oligotrophic or copiotrophic. This is also the case for the bacterial community of new soils lacking significant amounts of organic nutrients, such as the Mt. Merapi volcanic deposits of the present study. Furthermore, Castle *et al.* (6) analyzed microbial community convergence across actively retreating glaciers and reported that bacterial community differences strongly correlated with discriminative shifts in *Acidobacteria* (a relatively abundant member of mature forest soils that was present at a low abundance in young glacial soil). The opposite was true for *Betaproteobacteria*. To clarify the relationship between these groups in more detail, an attempt was made to calculate the ratio of *Betaproteobacteria* to *Acidobacteria* from the data of earlier studies on volcanic deposits, deglaciated soils, and other newly exposed substrates. As shown in Fig. 5, plots of the ratio against the corresponding TC or total organic carbon (TOC) appear to show a negative relationship approximated as *y*=0.76 *x*^−0.58^ ( *r*^2^=0.661), suggesting the abundance of *Betaproteobacteria* in lower carbon availability. As a reference, plots of the ratio of *Alphaproteobacteria* to *Acidobacteria* are not clearly dependent on TC or TOC (Fig. 5). As discussed above, the class *Betaproteobacteria* comprises various members that perform chemolithotrophic growth by using atmospheric trace gases and proliferates in association with pioneer mosses. Therefore, the ecological attributes of *Betaproteobacteria* appear to be important in early soil genesis and ecosystem development as well as in soils with high C availability (15).

In summary, the present results indicate that a phylogenetically diverse microbial community developed in Mt. Merapi volcanic deposits three years after the 2010 eruption and early colonists were mostly members of *Betaproteobacteria*. By comparing the bacterial communities on unvegetated and moss-covered deposits, we may assume that an inter-family succession of *Betaproteobacteria* occurred from chemolithotrophic to moss-associated groups. Further studies involving the isolation and characterization of culturable bacteria are now in progress to obtain more detailed information on bacterial contributions to early soil genesis.

## Supplementary material



## References

[1] Bansal S., Nilsson M., Wardle D.A. (2012). Response of photosynthetic carbon gain to ecosystem retrogression of vascular plants and mosses in the boreal forest. Oecologia.

[2] Bardgett R.D., Browman W.D., Kaufmann R., Schmidt S.K. (2005). A temporal approach to linking aboveground and belowground ecology. Trends Ecol Evol.

[3] Battaglia-Brunet F., Itard Y., Garrido F., Delorme F., Crouzet C., Greffié C., Joulian C. (2006). A simple biogeochemical process removing arsenic from a mine drainage water. Geomicrobiol J.

[4] Broady P., Given D., Greenfield L., Thompson K. (1987). The biota and environment of fumaroles on Mt. Melbourne, Northern Victoria Land. Polar Biol.

[5] Camus G.A., Gourgaud A., Mossand-Berthommier P.-C, Vincent P.-M (2000). Merapi (Central Java, Indonesia): An outline of the structural and magmatological evolution, with a special emphasis to the major pyroclastic events. J Volcanol Geotherm Res.

[6] Castle S.C., Nemergut D.R., Grandy A.S., Leff J.W., Graham E.B., Hood E., Schmidt S.K., Wickings K., Cleveland C.C. (2016). Biogeochemical drivers microbial community convergence across actively retreating glaciers. Soil Biol Biochem.

[7] Caudron C., Syahbana D.K., Lecocq T., Hinsberg V.V., McCausland W., Triantafyllou A., Camelbeeck T., Bernard A., Surono (2015). Kawah Ijen volcanic activity: a review. Bull Volcanol (Heidelberg).

[8] Clarkson B.R., Clarkson B.D. (1995). Recent vegetation changes on Mount Tarawera, Rotorua, New Zealand. NZ J Bot.

[9] Conrad R. (1996). Soil microorganism as controllers of atmospheric trace gases (H_2_, CO, CH_4_, OCS, N_2_O, and NO). Microbiol Rev.

[10] Cronin S.J., Lube G., Dayudi D.S., Sumarti S., Subrandiyo S. (2013). Insights into the October–November 2010 Gunung Merapi eruption (Central Java, Indonesia) from the stratigraphy, volume and characteristics of its pyroclastic deposits. J Volcanol Geotherm Res.

[11] Diaz-Herraiz M., Jurado V., Cueszva S., Laiz L., Pallecchi P., Tiano P., Sanchez-Moral S., Saiz-Jimenez C. (2013). The actinobacterial colonization of Etruscan paintings. Sci Rep.

[12] Drudge C.N., Warren L.A. (2014). Diurnal floc generation from neuston biofilms in two contrasting freshwater lakes. Environ Sci Technol.

[13] Eder W., Wanner G., Ludwig W., Busse H., Ziemke-Kägeler F., Lang E. (2011). Description of *Undibacterium oligocarboniphilum* sp. nov., isolated from purified water, and *Undibacterium pigrum* strain CCUG 49012 as the type strain of *Undibacterium parvum* sp. nov., and emended descriptions of the genus Undibacterium and the species *Undibacterium pigrum*. Int J Syst Evol Microbiol.

[14] Eyice O., Namura M., Chen Y., Mead A., Samavedam S., Schäfer H. (2015). SIP metagenomics identifies uncultivated *Methylophilaceae* as dimethylsulphide degrading bacteria in soil and lake sediment. ISME J.

[15] Fierer N., Bradford M.A., Jackson R.B. (2007). Toward an ecological classification of soil bacteria. Ecology.

[16] Fujimura R., Sato Y., Nishizawa T., Nanba K., Oshima K., Hattori M., Kamijo T., Ohta H. (2012). Analysis of early bacterial communities on volcanic deposits on the island of Miyake (Miyake-Jima), Japan: a 6-year study at a fixed site. Microbes Environ.

[17] Fujimura R., Kim S., Sato Y., Oshima K., Hattori M., Kamijo T., Ohta H. (2016). Unique pioneer microbial communities exposed to volcanic sulfur oxide. Sci Rep.

[18] Glöckner F.O., Babenzien H., Amann R. (1998). Phylogeny and identification in situ of *Nevskia ramosa*. Appl Environ Microbiol.

[19] Gomez-Alvarez V., King G.M., Nüsslein K. (2007). Comparative bacterial diversity in recent Hawaiian volcanic deposits of different ages. FEMS Microbiol Ecol.

[20] Grayston S.J., Wang S.Q., Campbell C.D., Edwards A.C. (1998). Selective influence of plant species on microbial diversity in the rhizosphere. Soil Biol Biochem.

[21] Green S.J., Inbar E., Michel F.C., Hadar Y., Minz D. (2006). Succession of bacterial communities during early plant development: transition from seed to root and effect of compost amendment. Appl Environ Microbiol.

[22] Green S.J., Michel F.C., Hadar Y., Minz D. (2007). Contrasting pattern of seed and root colonization by bacteria from the genus *Chryseobacterium* and from the family *Oxalobacteracae*. ISME J.

[23] Guo Y., Fujimura R., Sato Y., Suda W., Kim S., Oshima K., Hattori M., Kamijo T., Narisawa K., Ohta H. (2014). Characterization of early microbial communities on volcanic deposits along a vegetation gradient on the island of Miyake, Japan. Microbes Environ.

[24] He Z., Xie X., Xiao S., Liu J., Qiu G. (2007). Microbial diversity of mine water at Zhong Tiaoshan copper mine, China. J Basic Microbiol.

[25] Ibekwe A.M., Kennedy A.C., Halvorson J.J., Yang C.H. (2007). Characterization of developing microbial communities in Mt. St. Helens pyroclastic substrate. Soil Biol Biochem.

[26] Im W.T., Liu Q., Yang J., Kim M., Kim S., Lee S., Yi T. (2010). *Panacagrimonas perspica* gen. nov., sp. nov., a novel member of *Gammaproteobacteria* isolated from soil of ginseng field. J Microbiol.

[27] Jackson T.A. (2015). Weathering, secondary mineral genesis, and soil formation caused by lichens and mosses growing on granitic gneiss in a boreal forest environment. Geoderma.

[28] Ji M., Greening C., Vanwonterghem I. (2017). Atmospheric trace gases support primary production in Antarctic desert surface soil. Nature.

[29] Kalyuzhnaya M.G., Bowerman S., Lara J.C., Lidstrom M.E., Christoserdava L. (2006). *Methylotenera mobilis* gen. nov., sp. *Methylotenera mobilis* gen. nov., sp. nov., an obligately methylamine-utilizing bacterium within the family *Methylophilaceae*. Int J Syst Evol Microbiol.

[30] Kalyuzhnaya M.G., Beck D.A.C., Vorobev A., Smalley N., Kunkel D.D., Lidstrom M.E., Chistoserdova L. (2012). Novel methylotrophic isolates from lake sediment, description of *Methylotenera versatilis* sp. nov. and emended description of the genus *Methylotenera*. Int J Syst Evol Microbiol.

[31] Kamagata Y., Fulthorpe R.R., Tamura K., Takami H., Forney L.J., Tiedje J.M. (1997). Pristine environments harbor a new group of oligotrophic 2, 4-dichlorophenoxyacetic acid-degrading bacteria. Appl Environ Microbiol.

[32] Kaštovská K., Elster J., Stibal M., Šantrůcková H. (2005). Microbial assemblages in soil microbial succession after glacial retreat in Svalbard (high Arctic). Microb Ecol.

[33] Kelly L.C., Cockell C.S., Thorsteinsson T., Marteinsson V., Stevenson J. (2014). Pioneer microbial communities of the Fimmvöròuháls lava flow, Eyjafjallajökull, Iceland. Microb Ecol.

[34] Kim S.W., Suda W., Kim S., Oshima K., Fukuda S., Ohno H., Morita H., Hattori M. (2013). Robustness of gut microbiota of healthy adults in response to probiotic intervention revealed by high-throughput pyrosequencing. DNA Res.

[35] King G.M. (2003). Contributions of atmospheric CO and hydrogen uptake to microbial dynamics on recent Hawaiian volcanic deposits. Appl Environ Microbiol.

[36] King G.M. (2007). Chemolithotrohic bacteria: distributions, functions and significance in volcanic environments. Microbes Environ.

[37] Knelman J.E., Legg T.M., O’Neill S.P., Washenberger C.L., González A., Cleveland C.C., Nemergut D.R. (2012). Bacterial community structure and function change in association with colonizer plants during early primary succession in a glacier fore field. Soil Biol Biochem.

[38] Knight R., Maxwell P., Birmingham A. (2007). PyCogent: a toolkit for making sense from sequence. Genome Biol.

[39] Kojima H., Fukui M. (2011). *Sulfuritalea hydrogenivorans* gen. nov., sp. nov., a facultative autotroph isolated from a freshwater lake. Int J Syst Evol Microbiol.

[40] Kurata S., Kanagawa T., Yamada K., Torimura M., Yokomaku T., Kamagata Y., Kurane R. (2001). Fluorescent quenching-based quantitative detection of specific DNA/RNA using a BODIPY(R) FL-labeled probe or primer. Nucleic Acids Res.

[41] Lagerstrὂm A., Nilsson M-C, Zackrisson O., Wardle D.A. (2007). Ecosystem input of nitrogen through biological fixation in feather mosses during ecosystem retrogression. Funct Ecol.

[42] Land V., Skotnicki M.L., Selkirk P.M., Broady P., Adam K.D., Ninham J.A. (2001). Dispersal of the moss *Campylopus pyriformis* on geothermal ground near the summits of Mount Erebus and Mount Melbourne, Victoria Land, Antarctica. Antarct Sci.

[43] Lincoln S.P., Fermor T.R., Tindall B.J. (1999). *Janthinobacterium agaricidamnosum* sp. nov., a soft rot pathogen of *Agaricus bisporus*. Int J Syst Evol Microbiol.

[44] Lindo Z., Gonzalez A. (2010). The bryosphere: an integral and influential component of the earth’s biosphere. Ecosystems.

[45] Liu J., Kong W., Zhang G., Khan A., Guo G., Zhu C., Wei X., Kang S., Morgan-Kiss R.M. (2016). Diversity and succession of autotrophic microbial community in high-elevation soils along deglaciation chronosequence. FEMS Microbiol Ecol.

[46] Lu H., Fujimura R., Sato Y., Nanba K., Kamijo T., Ohta H. (2008). Characterization of *Herbaspirillum*- and *Limnobacter*-related strain isolated from young volcanic deposits in Miyake-Jima island, Japan. Microbes Environ.

[47] Nara K., Nakaya H., Wu B., Zhou Z., Hogetsu T. (2003). Underground primary succession of ectomycorrhizal fungi in a volcanic desert on Mount Fuji. New Phytol.

[48] Navarrete A.A., Venturini A.M., Meyer K.M., Klein A.M., Tiedje J.M., Bohannan B.J.M., Nüsslein K., Tsai S.M., Rodrigues J.L.M. (2015). Differential response of *Acidobacteria* subgroups to forest-to-pasture conversion their biogeographic patterns western. Brazilian Amazon Front Microbiol.

[49] Nemergut D.R., Anderson S.P., Cleveland C.C., Martin A.E., Miller E., Seimon A., Schmidt S.K. (2007). Microbial community succession in an unvegetated, recently deglaciated soil. Microb Ecol.

[50] Nishizawa T., Komatsuzaki M., Kaneko N., Ohta H. (2008). Archaeal diversity of upland rice field soils assessed by the terminal restriction fragment length polymorphism method combined with real time quantitative-PCR and a clone library analysis. Microbes Environ.

[51] Sato Y., Nishihara H., Yoshida M., Watanabe M., Rondal J.D., Ohta H. (2004). Occurrence of hydrogen-oxidizing *Ralstonia* species as primary Microorganisms in the Mt. Pinatubo volcanic mudflow deposits. Soil Sci Plant Nutr.

[52] Sato Y., Hirofumi N., Yoshida M., Watanabe M., Rondal J.D., Rogelio N., Ohta H. (2006). *Cupriavidus pinatubonensis* sp. nov. and *Cupriavidus laharis* sp. nov., novel hydrogen-oxidizing, facultatively chemolithotrophic bacteria isolated from volcanic mudflow deposits from Mt. Pinatubo in the Philippines. Int J Syst Evol Microbiol.

[53] Sato Y., Hosokawa K., Fujimura R., Nishizawa T., Kamijo T., Ohta H. (2009). Nitrogenase activity (acetylene reduction) of an iron-oxidizing *Leptospirillum* strain cultured as a pioneer microbe from a recent volcanic deposit on Miyake-Jima, Japan. Microbes Environ.

[54] Schmidt S.K., Reed S.C., Nemergut D.R. (2008). The earliest stages of ecosystem succession in high-elevation (5000 metres above sea level), recently deglaciated soils. Proc Biol Sci.

[55] Street L.E., Subke J-A., Sommerkorn M., Sloan V., Ducrotoy H., Phoenix G.K., Williams M. (2013). The role of mosses in carbon uptake and partitioning in arctic vegetation. New Phytol.

[56] Summers S., Whiteley A.S., Kelly L.C., Cockell C.S. (2013). Land coverage influences the bacterial community composition in the critical zone of a sub-Arctic basaltic environment. FEMS Microbiol Ecol.

[57] Surono M., Jousset P., Pallister J. (2012). The 2010 explosive eruption of Java’s Merapi volcano—a 100 years event. J Volcanol Geotherm Res.

[58] Suyama T., Tokiwa Y., Ouichanpagdee P., Kanagawa T., Kamagata Y. (1998). Phylogenetic affiliation of soil bacteria that degrade aliphatic polyesters available commercially as biodegradable plastics. Appl Environ Microbiol.

[59] Tani A., Akita M., Murase H., Kimbara K. (2011). Culturable bacteria in hydrophonic cultures of moss *Racomitrum japonicum* and their potential as biofertilizers for moss production. J Biosci Bioeng.

[60] Uroz S., Calvaruso C., Turpault M.P., Sarniguet A., de Boer W., Leveau J.H.J., Frey-Klett P. (2009). Efficient mineral weathering is a distinctive functional trait of the bacterial genus *Collimonas*. Soil Biol Biochem.

[61] Uroz S., Calvaruso C., Turpault M.P., Frey-Klett P. (2009). Mineral weathering by bacteria: ecology, actors, and mechanisms. Trends Microbiol.

[62] Watanabe T., Kojima H., Fukui M. (2015). *Sulfuriferula multivorans* gen. nov., sp. nov., isolated from a freshwater lake, reclassification of ‘*Thiobacillus plumbophilus*’ as *Sulfuriferula plumbophilus* sp. nov., and description of *Sulfuricellaceae* fam. nov. and *Sulfuricellales* ord. nov. Int J Syst Evol Microbiol.

[63] Watanabe T., Kojima H., Fukui M. (2016). *Sulfuriferula thiophila* sp. nov., a chemolithoautotrophic sulfur-oxidizing bacterium, and correction of the name *Sulfuriferula plumbophilus* to *Sulfuriferula plumbiphila* corrig. Int J Syst Evol Microbiol.

[64] Watts S.F. (2000). The mass budgets of carbonyl sulfide, dimethyl sulfide, carbon disulfide and hydrogen sulfide. Atmos Environ.

[65] Weber C.F., King G.M. (2010). Distribution and diversity of carbon monoxide-oxidizing bacteria and bulk bacterial communities across a succession gradient on a Hawaiian volcanic deposit. Environ Microbiol.

[66] Weber C.F., King G.M. (2010). Quantification of Burkholderia coxL genes in Hawaiian volcanic deposits. Appl Environ Microbiol.

[67] Weber C.F., King G.M. (2017). Volcanic Soils as sources of novel CO-oxidizing *Paraburkholderia* and *Burkholderia*: *Paraburkholderia hiiakae* sp. nov., *Paraburkholderia metrosideri* sp. nov., *Paraburkholderia paradisi* sp. nov., *Paraburkholderia peleae* sp. nov., and *Burkholderia alpina* sp. nov. a member of the *Burkholderia cepacia* complex. Front Microbiol.

[68] Wulaningsih T., Humaida H., Harijoko A., Koichiro W. (2013). Major element and rare earth elements investigation of Merapi Volcano, Central Java, Indonesia. Procedia Earth Planet Sci.

[69] Yoshitake S., Fujiyoshi M., Watanabe K., Masuzawa T., Nakatsubo T., Koizumi H. (2012). Successional changes in the soil microbial community along vegetation development sequence in a subalpine volcanic desert on Mount Fuji, Japan. Plant Soil.

[70] Yoshitake S., Uchida M., limura Y., Ohtsuka T., Nakatsubo T. (2018). Soil microbial succession along a chronosequence on a High Arctic glacier foreland, Ny-Ålesund, Svalbard: 10 years’ change. Polar Sci.

[71] Zhou J., Bruns M.A., Tiedje J.M. (1996). DNA recovery from soils of diverse composition. Appl Environ Microbiol.

