# Supplementary material for: Comparative Characterization of Bacterial Communities in Moss-Covered and Unvegetated Volcanic Deposits of Mount Merapi, Indonesia
Source: Microbes Environ. 2019 Jul 20;34(3):268–77. doi: 10.1264/jsme2.ME19041 (PMC6759343; doi:10.1264/jsme2.ME19041)
Supplement: Supplementary file 1 [file 34_268_s1.pdf]

Supplementary material

Comparative characterization of bacterial communities in moss-covered and unvegetated volcanic deposits of Mount Merapi, Indonesia

ANNISA N. LATHIFAH[1,2], YONG GUO[2], NOBUO SAKAGAMI[1,2], WATARU SUDA[3], MASANOBU HIGUCHI[4], TOMOYASU NISHIZAWA[1,2], IRFAN D. PRIJAMBADA[5], and HIROYUKI OHTA[1,2]

[1]*United Graduate School of Agricultural Science, Tokyo University of Agriculture and Technology, 3-5-8 Saiwai-cho, Fuchu-shi, Tokyo 183-8509, Japan*
[2] *Ibaraki University College of Agriculture, 3-21-1 Chuo, Ami-machi, Ibaraki 300-0393, Japan*
[3]*Department of Computational Biology, Graduate School of Frontier Science, The University of Tokyo, Kashiwa, Japan*
[4] *Department of Botany, National Museum of Nature and Science, 4-1-1, Amakubo, Ibaraki, Japan*
[5]*Graduate School of Biotechnology, University of Gadjah Mada, Yogyakarta, Indonesia*

Table S1. Chemical properties of the Mt. Merapi volcanic deposit (BRD, BRUD) and forest soil (FRS) samples*.

Sample	pH	Total C ($g\ kg^{-1}$)	Total N ($g\ kg^{-1}$)	C:N ratio	Water content (%)
BRD	7.3 ± 0.2^a	0.09 ± 0.01^a	0.01 ± 0.00^a	5.1 ± 0.45^b	4.4 ± 0.2^a
BRUD	7.5 ± 0.02^a	0.04 ± 0.00^a	0.01 ± 0.00^a	2.3 ± 0.25^a	5.7 ± 0.2^a
FRS	7.6 ± 0.2^a	25.8 ± 1.81^b	2.10 ± 0.26^b	12.3 ± 0.75^c	21.3 ± 3.1^b

* Each value is expressed as the mean and SD of triplicate samples. Different letters in the same column indicate significant differences between values within a given comparison (ANOVA with Tukey's HSD test, $P < 0.05$)

Table S2. Number of OTUs and diversity indices of bacterial communities based on pyrosequencing data in the volcanic deposit (BRD, BRUD) and forest soil (FRS) samples of Mt. Merapi*.

**Sample**	**Number of OTUs**	**Diversity indices**		
		Shannon	Inverse Simpson	ChaoI
BRD	396 ± 50^b	5 ± 0.35^a	72.78 ± 46.37^a	803 ± 101^b
BRUD	287 ± 6^a	4.35 ± 0.28^a	33.22 ± 15.49^a	531 ± 21^a
FRS	751 ± 47^c	6.16 ± 0.06^b	278.43 ± 28.47^b	1627 ± 138^c

* An OTU was defined as a group of sequences with similarity of 96% or more. Each value is expressed as the mean and SD of triplicate samples. Different letter indicates significant differences between mean values within a given comparison (ANOVA with Tukey's HSD test, $P<0.05$)

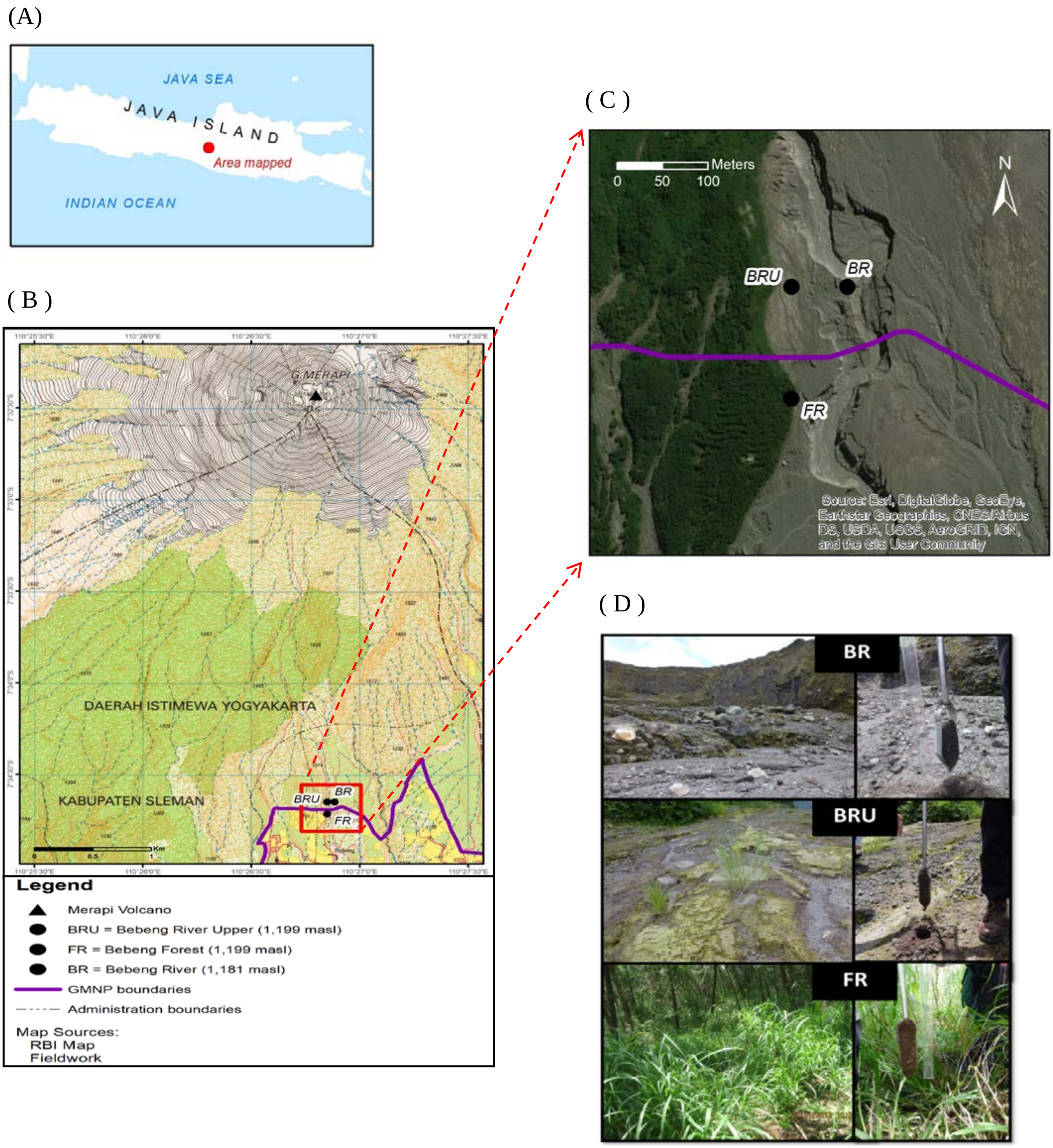

Fig. S1. Maps and photographs of the study/sampling sites. (A) Map showing the location of Mt. Merapi (volcano) in Central Java; (B) The study sites in the north of Yogyakarta; (C) Satellite imagery of the study sites (green, forest/vegetated area; grey, unvegetated area); (D) Photographs of sampling sites taken in 2013. Map A adapted from official website of "Balai Penyelidikan dan Pengembangan Teknologi Kebencanaan Geologi", Ministry of Energy and Mineral Resources, Yogyakarta, Indonesia (http://merapi.bgl.esdm.go.id/pub/page.php?idx=358) and Maps B and C from Dwita Nur Restiani, M. Sc at Department of Geoinformation for spatial planning and disaster risk management, UGM.

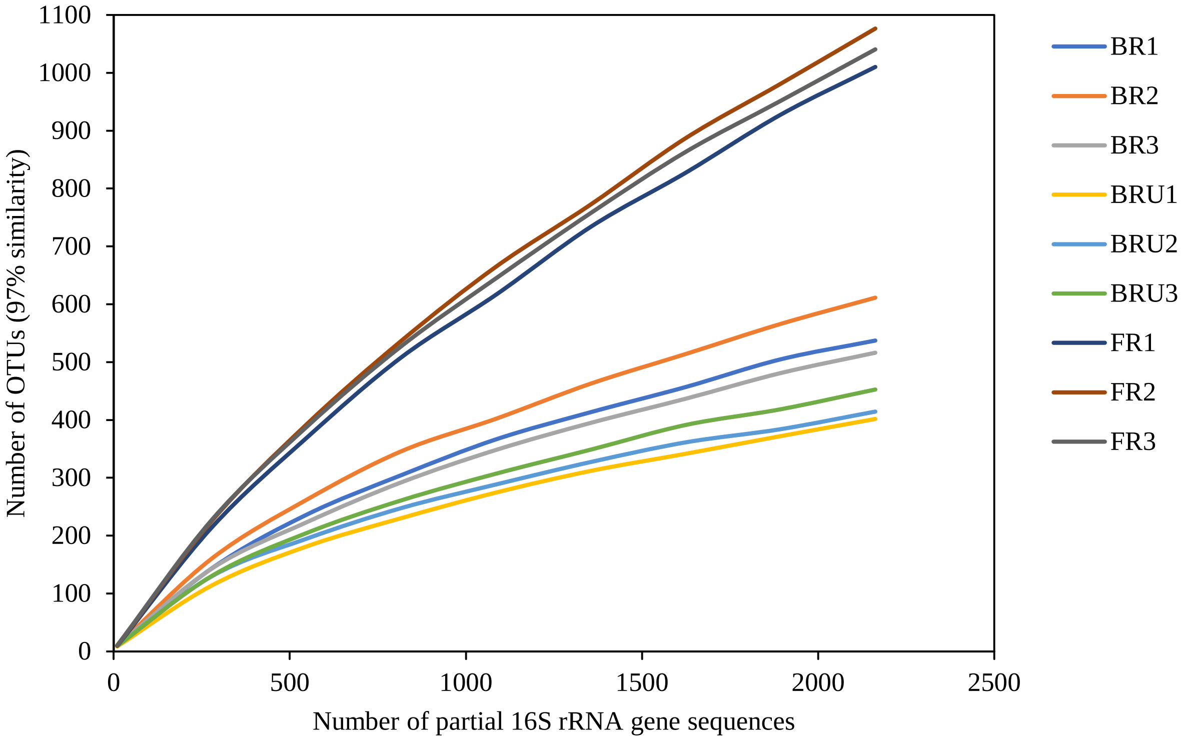

Fig. S2. Rarefaction curves for the bacterial communities of Mt. Merapi volcanic deposits (BR1-3 and BRU1-3) and the forest soil (FR1-3) samples based on tag-pyrosequencing of 16S rRNA genes. Curves are shown at 97% sequence similarity.

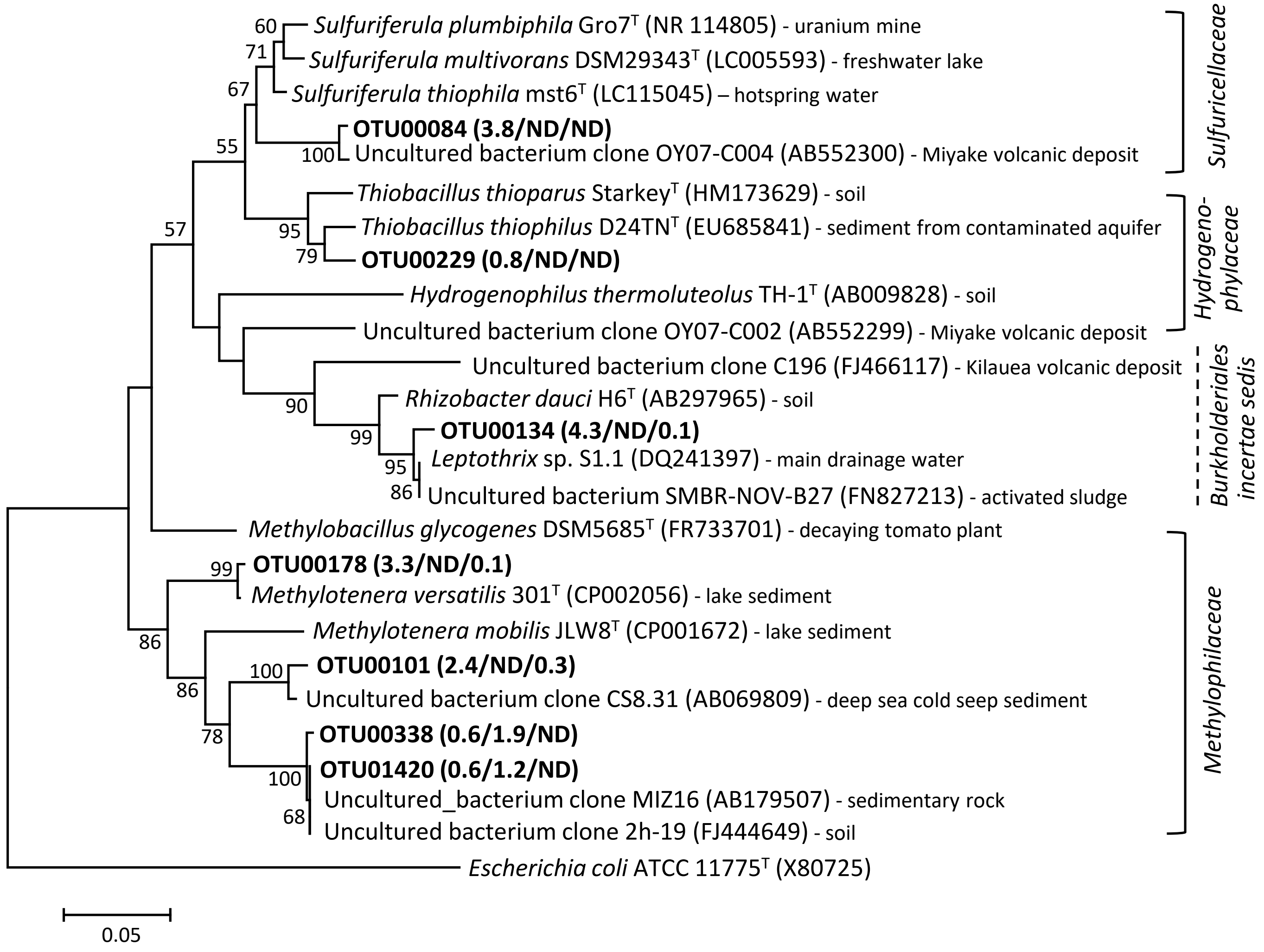

Fig. S3. Neighbor-joining phylogenetic tree based on 16S rRNA gene partial sequences (279 nucleotides length) shows the positions of OTUs from Mt. Merapi samples (Merapi OTUs) in the families: *Hydrogenophylaceae*, *Methylophilaceae*, *Sulfuricellaceae*, and *Burkholderiales incertae sedis* are shown. The 16S rRNA coding sequence of *Escherichia coli* ATCC 11775^{T} was observed as an outgroup. Numbers on branch nodes are the bootstrap values (>50%) expressed as a percentage (*n*=1000). Bar: 0.05 substitution per nucleotide position. Number in parentheses after each OTU gives the relative abundance (in percentage; ND, <0.1%) of the OTU in the BRD, BRUD, and FRS communities, respectively. The sources of strains and clones are written after the accession number of the taxa.

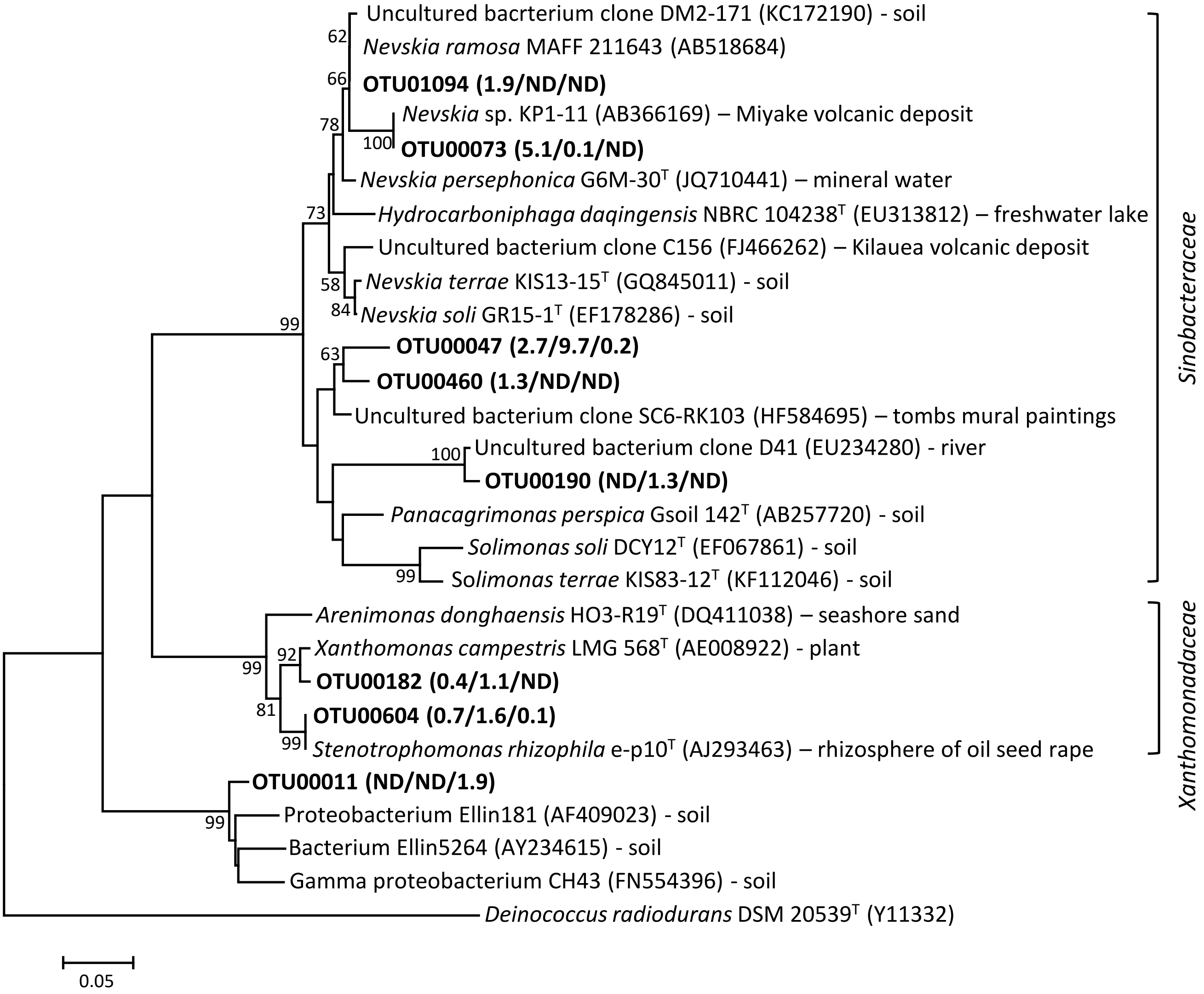

Fig. S4. Neighbor-joining phylogenetic tree based on 16S rRNA gene partial sequences (241 nucleotides length) shows the positions of the Merapi OTUs in *Sinobacteraceae* and *Xanthomonadaceae*. The 16S rRNA coding sequence of *Deinococcus radiodurans* DSM 20539^T was observed as an outgroup. Numbers on branch nodes represent bootstrap values (>50%) expressed as a percentage (*n*=1000). Bar: 0.05 substitution per nucleotide position. Number in parentheses after each OTU gives the relative abundance (in percentage; ND, <0.1%) of the OTU in the BRD, BRUD, and FRS communities, respectively. The sources of strains and clones are written after the accession number of the taxa.

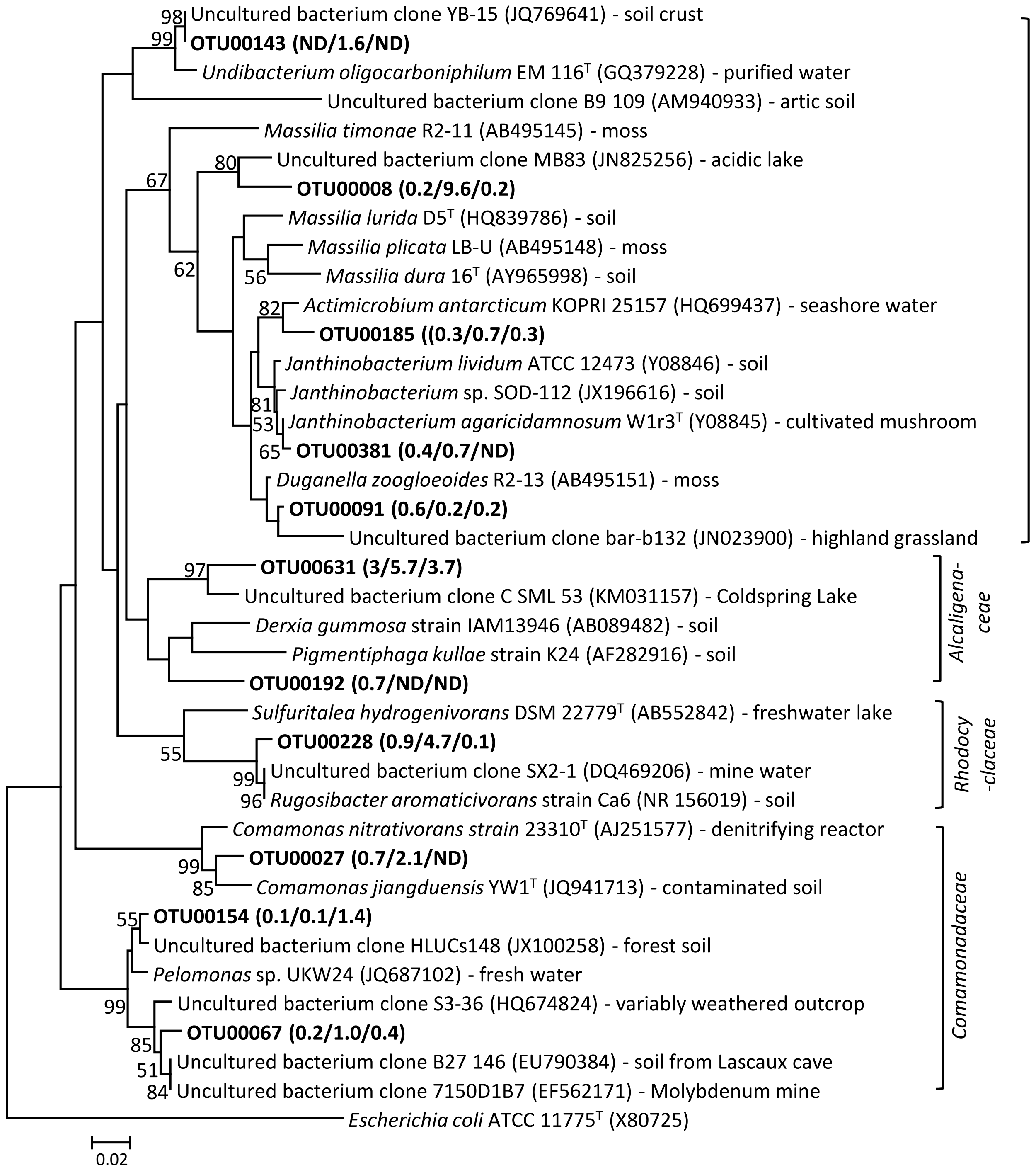

Fig. S5. Neighbor-joining phylogenetic tree based on 16S rRNA gene partial sequences (294 nucleotides length) shows the position of Merapi OTUs in the families *Alcaligenaceae*, *Comamonadaceae*, *Oxalobacteraceae*, and *Rhodocyclaceae*. The 16S rRNA coding sequence of *Escherichia coli* ATCC 11775^{T} was observed as an outgroup. Numbers on the branch nodes represent bootstrap values (>50%) expressed as a percentage (n=1000). Bar: 0.02 substitution per nucleotide position. Number in parentheses after each OTU gives the relative abundance (in percentage; ND, <0.1%) of the OTU in the BRD, BRUD, and FRS communities, respectively.

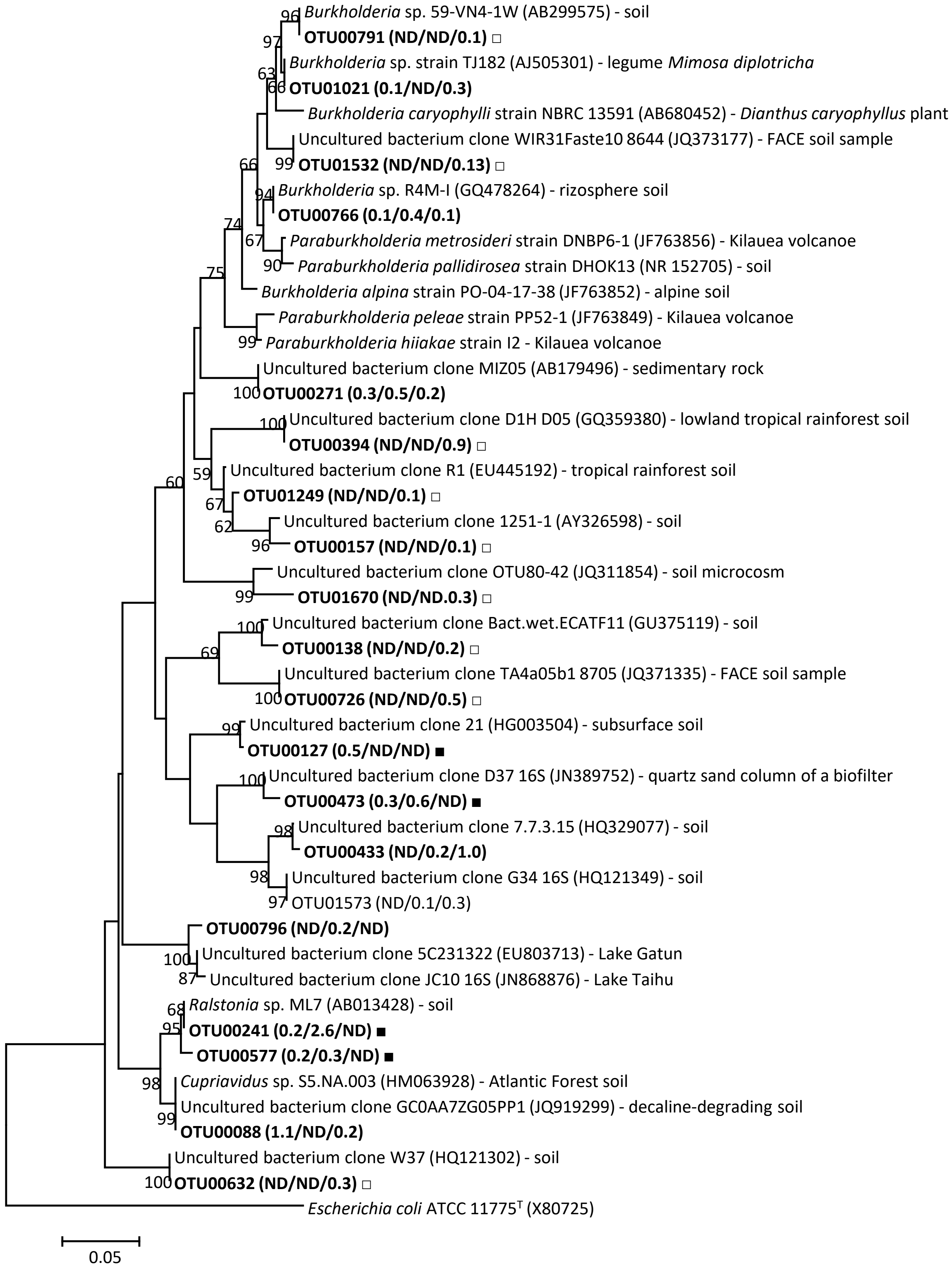

Fig. S6. Neighbor-joining phylogenetic tree based on 16S rRNA gene the partial sequences (245 nucleotides length) shows the positions of Merapi OTUs in *Burkholderiaceae*. The 16S rRNA coding sequence of *Escherichia coli* ATCC 11775^T was noted as an outgroup. Numbers on branch nodes are the bootstrap values (>50%) expressed as a percentage (n=1000). Bar: 0.05 substitution per nucleotide position. Number in parentheses after each OTU gives the relative abundance (in percentage; ND, <0.1%) of the OTU in the BRD, BRUD, and FRS communities, respectively. Symbols: ■, OTUs specific to BRD and BRUD; □, OTUs specific to FRS.

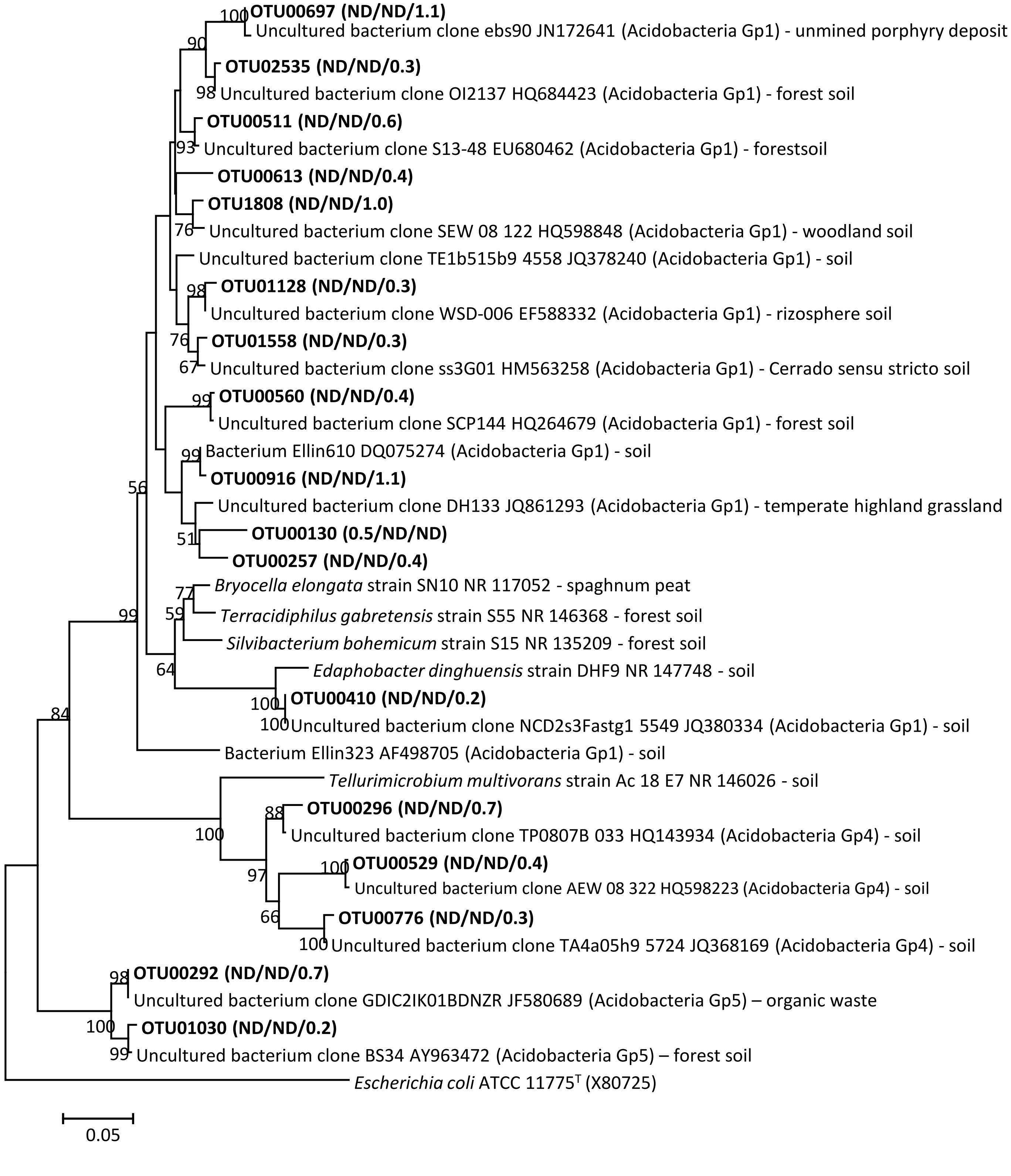

Fig. S7. Neighbor-joining phylogenetic tree based on 16S rRNA gene partial sequences (326 nucleotides length) gives the position of Merapi OTUs in *Acidobacteriaceae* and undefined groups. The 16S rRNA coding sequence of *Escherichia coli* ATCC 11775^T was observed as an outgroup. Numbers on branch nodes are the bootstrap values (>50%) expressed as a percentage (n=1000). Bar: 0.05 substitution per nucleotide position. Number in parentheses after each OTU gives the relative abundance (in percentage; ND, <0.1%) of the OTU in the BRD, BRUD, and FRS communities, respectively.
